# The Efficacy of Japanese Herbal Kampo Medicine as an Acute and Prophylactic Medication to Treat Chronic Daily Headache and Medication Overuse Headache:-Single Arm Retrospective Study

**DOI:** 10.7759/cureus.25419

**Published:** 2022-05-27

**Authors:** Masahito Katsuki, Kenta Kashiwagi, Shin Kawamura, Akihito Koh

**Affiliations:** 1 Department of Neurosurgery, Itoigawa General Hospital, Itoigawa, JPN; 2 Department of Neurology, Itoigawa General Hospital, Itoigawa, JPN

**Keywords:** primary headache disorder, kakkonto (tj-1), goshuyuto (tj-31), goreisan (tj-17), alternative medical therapy, medication-overuse headache (moh), japanese herbal kampo medicine, chronic tension-type headache (ctth), chronic migraine (cm), chronic daily headache (cdh)

## Abstract

Introduction

A chronic daily headache (CDH) comprises a group of headaches occurring at least 15 days per month for three or more consecutive months. We retrospectively investigated the effectiveness of the hybrid treatment strategy for CDH using *Kampo* medicine combined with Western medication.

Methods

We retrospectively investigated 43 consecutive first-visit CDH patients. In addition to Western acute and prophylactic medications, we prescribed three types of *Kampo* medicines: *goreisan*, *goshuyuto*, and *kakkonto* depending on the patients’ symptoms. Headache impact test-6 (HIT-6), monthly headache days (MHD), monthly migraine days (MMD), and monthly acute medication intake days (AMD) before, 1- and 3-months after starting the hybrid medications were assessed as outcomes.

Results

Thirty-six women and seven men were included. The median age was 51 years old. Nine were chronic migraine (CM), 22 were episodic migraine and tension-type headaches (EM+TTH), and 12 were chronic TTH. Twenty-seven patients also had medication overuse headaches (MOH). The medians of HIT-6 before, one and three months after treatment were 63, 48, and 40, respectively. Those of MHD were 20, 5, and 2. Those of MMD were 2, 0, and 0. Those of AMD were 15, 0, and 0. Significant reductions in HIT-6, MDH, MMD, and AMD were observed one and three months after starting *Kampo *treatment. Similar trends were observed in the EM+TTH and MOH patients as subgroup analyses.

Conclusion

The hybrid medication strategy of *Kampo *and Western medicines for CDH is safe and effective in terms of both acute and prophylactic medications with rapid efficacy.

## Introduction

A chronic daily headache (CDH) comprises a group of headaches occurring at least 15 days per month for three or more consecutive months. About 5% of the normal population suffers from CDH [1]. The treatment for CDH is difficult because (1) acute medications are ineffective in 25% of patients, which can cause a transformation from episodic to chronic headache [2], medication-overuse headache (MOH) [3,4], and other side effects [5], and (2) prophylactic medications are ineffective in about 50% of patients and have side effects, leading to poor patient adherence, with more than half of the patients stopping treatment within two months [6]. In this context, alternative acute and prophylactic medications for CDH are needed. The ideal medication is a drug that can be used for headache attacks, may not lead to chronic headache and MOH, and can also be used prophylactically with rapid effectiveness.

In Japan, to solve these problems of Western medicine, Japanese traditional herbal *Kampo* medicine can be used for headache treatment and is also described in the Japanese Clinical Practice Guideline for Headache 2021 [7]. *Kampo* medicine can be used as both acute [8] and prophylactic therapy [9-12] for headaches. In our hospital, we treated CDH patients with the so-called "hybrid medication strategy of *Kampo* and Western medicines" [13]. We retrospectively investigated the effectiveness of the CDH treatment strategy using *Kampo* medicine, including goreisan, goshuyuto, and kakkonto, combined with Western medication.

## Materials and methods

Study population

From the medical records between October 2021 and May 2022, we retrospectively investigated 43 consecutive first-visit CDH patients who presented at our headache-specialized outpatient. All the patients suffered from headaches at least 90 days before the first visit and the *Kampo* treatment. The headache diagnosis was based on the International Classification of Headache Disorders, 3rd edition (ICHD-3) [14]. Chronic migraine (CM), episodic migraine with tension-type headache (EM+TTH), chronic TTH (CTTH), and MOH were diagnosed.

The hospital’s research ethics committee approved this study (approval number 2021-4), and we gained written informed consent for this study from all the patients or patients’ families. This retrospective study was performed following the Declaration of Helsinki.

Treatment strategy

After diagnosing the headache based on the ICHD-3, we treated the patients by referring to the Japanese Clinical Practice Guideline for Headache 2021 [7]. Depending on the severity, we prescribed acute medications such as non-steroidal anti-inflammatory drugs (NSAIDs) and triptans. In addition, we also prescribed prophylactic medications, such as lomerizine, propranolol, angiotensin receptor blockers, valproic acid, antidepressants, monoclonal calcitonin gene-related peptide antibodies (CGRP mAbs), and muscle relaxants. If specific prophylactic medications had been used in the past and were ineffective or had side effects, they were not to be prescribed aggressively.

We also prescribed three types of *Kampo* extract formulations considering the patients’ symptoms. The choice of a specific Kampo medicine was based on the guidelines [7], experts’ opinions [15], and previous Japanese reports [7,8,16-18]. For CDH patients with mainly TTH, we prescribed kakkonto [19]. In the case of CDH with migraine, we used two different drugs: goshuyuto for migraines with or without aura [9,20], or for those with sensitivity to cold or the menopausal disorder [21], and goreisan for patients with edema or dehydration (sudoku status; unbalance of water distribution in *Kampo* medicine theory) [22] or migraines associated with weather conditions [23,24]. All *Kampo* medications were taken as needed, depending on the patients’ symptoms. Multiple *Kampo* medicines were sometimes prescribed, and patients were given a choice depending on the characteristics of their headaches.

Notably, we instructed that the *Kampo* medicine could be taken prophylactically before a headache occurs or during prodrome symptoms. Besides, *Kampo* medicine could be taken daily against a headache as a prophylactic medication, with a maximum of three packets per day, in addition to the prescribed Western medicines. We also told the patients that *Kampo* medicine could be used as an acute medication when the headache was present but not so severe as to use NSAIDs or triptans. When a single *Kampo* intake could not resolve the headache, *Kampo* medicine could be used in combination with NSAIDs and triptans. This prescription policy, for *Kampo* medicine as both acute and prophylactic medication, was based on the fact that *Kampo* medicine contains a wide variety of substances, not a single active ingredient, and each component acts comprehensively on the entire body to safely produce a therapeutic effect [15]. Such a prescription policy of *Kampo* medicine as both acute and prophylactic medication is beginning to be widely practiced in Japan [13].

Clinical variables and outcomes

We collected patients’ characteristics, such as age, sex, comorbidities, and the onset of the headache (years ago). Clinical data reported by paper-based or electronic headache diaries were used. Monthly headache days (MHD), monthly migraine days (MMD), and monthly acute medication intake days (AMD) were defined as the monthly values over the respective observation period of 30 days. A headache day was defined as a day with any kind of headache; a migraine day was defined by patients when they had severe pain, migraine pain characteristics (pulsating, one-sided pain), aura symptoms, vegetative symptoms like phono- or photophobia, nausea, vomiting, need for rest, or when triptans were taken [25]. Headache impact test-6 (HIT-6) [26] was also investigated over the respective observation period. Monthly use days of acute medication and *Kampo* medicine were also collected from the headache diary. The prescribed prophylactic medications we started with *Kampo* medicine as the hybrid treatment were also checked. The outcomes were defined as the changes in HIT-6, MHD, MMD, and AMD before treatment and after one or three months.

Statistical Analysis

Results were presented as the median (range). A Friedman's test and a subsequent Wilcoxon’s test were performed to compare HIT-6, MHD, MMD, and AMD before treatment and after one or three months. We conducted these analyses using version 28.0.0 of SPSS software (IBM, NY, USA). A two-tailed p<0.05 was considered statistically significant. Bonferroni’s correction for multiple comparisons in each test was applied, but we did not apply it throughout the study [27].

## Results

General characteristics

Table 1 shows the characteristics of 43 CDH patients. Thirty-six women and seven men were included. The median age was 51 (15-99) years old. Of the 43 patients, 9 were CM, 22 were EM+TTH, and 12 were CTTH. Twenty-seven patients also had MOH. The median past years from the first repetitive headache was 20 (1-70) years. Goreisan, goshuyuto, and kakkonto were prescribed depending on the patients’ headache characteristics. Twenty-six patients also had prophylactic medications. The median use days of goreisan in the first month of treatment were 15 (0-30) days, those of goshuyuto were 4 (0-30) days, and those of kakkonto were 5 (0-30) days, with a maximum intake of three packets per day. Other details and characteristics of each headache type were also described in Table 1.

**Table 1:** Characteristics of the chronic daily headache patients The results are shown with the number (%) or the median (range). CM: chronic migraine, CTTH: chronic tension-type headache, CGRP mAbs; monoclonal calcitonin gene-related peptide antibodies, EM+TTH: episodic migraine with tension-type headache, MOH: medication-overuse headache, NSAIDs: non-steroidal anti-inflammatory drugs, HIT-6: head impact test-6, MHD: monthly headache day, MMD: monthly migraine day, AMD: acute medication intake day.

Variables	Total (n=43)	CM (n=9)	EM+TTH (n=22)	CTTH (n=12)	MOH (n=27)
Age (years old)	51 (15-99)	48 (15-57)	43 (15-74)	79 (40-99)	52 (15-99)
Women: men (%women)	36:7 (83.7%)	9:0 (100%)	19:3 (77.3%)	8:4 (66.7%)	23:4 (85.2%)
With medication overuse headache	27 (62.8%)	7 (77.8%)	11 (50.0%)	9 (75.0%)	27 (100%)
Monthly acute medication intake day before* Kampo* treatment					
Triptan	2 (0-30)	7 (0-30)	0 (0-8)	-	3 (0-30)
NSAIDs	7 (0-30)	12 (0-30)	2 (0-30)	0 (0-30)	9 (0-30)
Combination analgesic (most over-the-counter drugs containing two types of NSAIDs and caffeine)	7 (0-30)	4 (0-30)	0 (0-20)	13 (0-30)	10 (0-30)
Comorbidities					
Hypertension	7 (16.3%)	0	3 (13.6%)	4 (33.3%)	6 (22.2%)
Diabetes	2 (4.7%)	0	1 (4.5%)	1 (8.3%)	0
Dementia	4 (9.3%)	0	0	4 (33.3%)	3 (11.1%)
Back pain and knee pain	3 (7.0%)	0	2 (9.1%)	1 (8.3%)	1 (3.7%)
Premenstrual syndrome	3 (7.0%)	2 (22.2%)	1 (4.5%)	0	2 (7.4%)
Psychological disorders (depression, anxiety)	7 (16.3%)	2 (22.2%)	1 (4.5%)	4 (33.3%)	5 (18.5%)
Others (asthma, Basedow's disease, epilepsy, irritable bowel syndrome)	4 (9.3%)	3 (33.3%)	1 (4.5%)	0	3 (11.1%)
Onset (years ago) (n=39)	20 (1-70)	25 (5-45)	18 (1-48)	30 (1-70) (n=8)	20 (1-70) (n=23)
*Kampo* medicine (use days in the first month after starting* Kampo* treatment)					
Goreisan	15 (0-30)	20 (0-30)	16 (0-30)	9 (0-30)	16 (0-30)
Goshuyuto	4 (0-30)	6 (0-30)	0 (0-30)	2 (0-30)	4 (0-30)
Kakkonto	5 (0-30)	-	0 (0-30)	5 (0-30)	3 (0-40)
Prophylactic medications except for* Kampo* medicine					
Nothing	17 (39.5%)	0	10 (45.5%)	7 (5.8%)	6 (22.2%)
Lomerizine	3 (7.0%)	1 (11.1%)	1 (4.5%)	1 (8.3%)	3 (11.1%)
Propranolol	6 (14.0%)	2 (22.2%)	3 (13.6%)	1 (8.3%)	6 (22.2%)
Angiotensin receptor blocker	2 (4.7%)	0	1 (4.5%)	1 (8.3%)	2 (7.4%)
Valproic acid	6 (14.0%)	3 (33.3%)	2 (9.1%)	1 (8.3%)	5 (18.5%)
Antidepressant	4 (9.3%)	1 (11.1%)	2 (9.1%)	1 (8.3%)	3 (11.1%)
CGRP mAbs	3 (7.0%)	1 (11.1%)	2 (9.1%)	0	2 (7.4%)
Muscle relaxant	2 (4.7%)	1 (11.1%)	1 (4.5%)	0	0
HIT-6					
Pre (0 month)	63 (44-78)	66 (64-78)	60 (40-74)	59 (48-78)	65 (48-78)
1 month	48 (36-78)	50 (48-60)	48 (36-63)	45 (36-78)	48 (36-78)
3 months	40 (36-78)	44 (36-62)	38 (36-60)	37 (35-78)	40 (35-78)
MHD					
Pre (0 month)	20 (15-30)	23 (15-30)	16 (15-30)	30 (16-30)	20 (15-30)
1 month	5 (0-30)	8 (2-30)	7 (0-30)	5 (0-30)	5 (0-30)
3 months	2 (0-30)	3 (0-18)	3 (0-30)	0 (0-30)	2 (0-30)
MMD					
Pre (0 month)	2 (0-16)	12 (10-16)	4 (0-5)	-	4 (0-15)
1 month	0 (0-7)	1 (0-6)	0 (0-7)	-	0 (0-6)
3 months	0 (0-5)	0 (0-5)	0 (0-4)	-	0 (0-5)
Monthly AMD					
Pre (0 month)	15 (0-30)	15 (10-30)	12 (2-30)	30 (0-30)	16 (10-30)
1 month	0 (0-30)	2 (0-6)	0 (0-7)	1 (0-30)	1 (0-30)
3 months	0 (0-30)	1 (0-5)	0 (0-30)	0 (0-30)	0 (0-30)

Treatment response of *Kampo* medicine for CDH

Of all the 43 CDH patients, the median HIT-6 before, one and three months after treatment were 63 (44-78), 48 (36-78), and 40 (36-78), respectively. MHD before, one and three months after treatment were 20 (15-30), 5 (0-30), and 2 (0-30), respectively. Those about MMD were 2 (0-16), 0 (0-7), and 0 (0-5). Those about AMD were 15 (0-30), 0 (0-30), and 0 (0-30).

**Figure 1:**
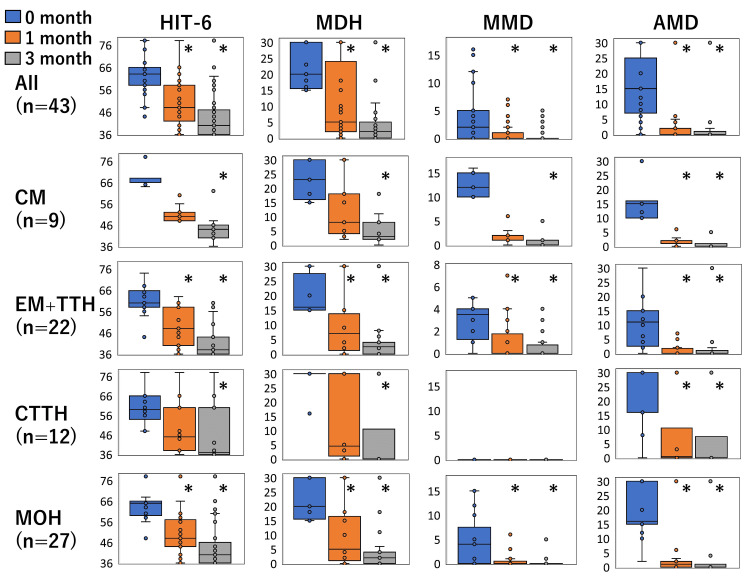
Treatment response Treatment response of *Kampo* medicine for chronic daily headache containing all the types of headaches, chronic migraine (CM), episodic migraine with tension-type headache (EM+TTH), chronic tension-type headache (CTTH), and medication-overuse headache (MOH). Boxplots of the headache impact test-6 (HIT-6), monthly headache days (MHD), monthly migraine days (MMD), and monthly acute medication intake days (AMD) (y-axis) before treatment (0 month), after one and three months of treatment (x-axis) were shown. HIT-6, MDH, MMD, and AMD were significantly improved after treatment (*; significantly decreased compared to 0 month adjusted by the Bonferroni’s correction, p < 0.01).

Regarding all the 43 CDH patients, significant reductions in HIT-6, MDH, MMD, and AMD were observed one month after starting *Kampo* treatment. These trends were also confirmed after three months. Similar trends were observed in the 22 EM+TTH patients and 27 MOH patients. In the 9 CM patients and 12 CTTH patients, a significant decrease in AMD at one and three months were observed, but those of HIT-6, MDH, and MMD were confirmed only at three months (Figure 1). Laboratory tests and physical examinations over three months confirmed no side effects of *Kampo* medicines, such as liver dysfunction, interstitial pneumonia, and pseudoaldosteronism.

## Discussion

We herein describe the results of our hybrid therapy for CDH using *Kampo* and Western medicines. *Kampo* medicine may act as both acute and prophylactic medications, leading to the rapid decrease of HIT-6, MHD, MMD, and AMD, especially in EM+TTH and MOH patients. In addition, there were no side effects from *Kampo* medicine.

Treatment strategy for CDH

The standard CDH treatment is not established. However, the main treatment strategies are (1) to discontinue the current regimen of analgesic medications, (2) to rotate and select appropriate analgesic medicine specific to the headache characteristics, and (3) to initiate a prophylactic medication regimen to reduce the frequency and intensity of both the chronic headaches and the acute exacerbations [28].

About (1) and (2), in approximately 77% of CDH patients, discontinuation of the overused medication alone will result in the return to an episodic form of headache that can then be more easily managed [29]. However, some patients have withdrawal headaches or continuous headaches during this process, and bridging analgesics are needed [7,30]. Therefore, our results show that AMD and HIT-6 decreased rapidly by using *Kampo* medicine as an alternative acute medication [8] can help resolve the withdrawal headache or continuous headache and stop the medication overuse.

About (3), the consideration of the duration of prophylactic therapy as well as tapering and discontinuation of the therapy also depends on the severity of headache-induced disability before the prophylactic therapy, and no uniform criteria can be applied. However, it takes at least two months before the effectiveness of prophylactic therapy can be evaluated [7,31]. While determining efficacy during the two months, it is possible that CDH could recur. Some medications act rapidly, such as amitriptyline [32] and CGRP mAbs [25,33]. *Kampo* medicine can be a prophylactic medication that rapidly acts [7,18] and has long-term effects [10]. Furthermore, *Kampo* medicine does not have a side effect of drowsiness, which is often confirmed by using amitriptyline. Also, *Kampo* medicine is not as expensive as CGRP mAbs. Our results showed the possibility that *Kampo* medicine can be prescribed as a prophylactic medication for CDH.

*Kampo* medicine for headache

*Kampo* medicine is empirically used for headache treatment. However, it cannot be denied that scientific evidence, such as basic and clinical research, remains insufficient. In addition to goreisan, goshuyuto, and kakkonto, keishininjinto, and chotosan are introduced in the Japanese guidelines [7]. Many other *Kampo* medicines are also prescribed for headache treatment. Therefore, further studies with a strong evidence level should be needed, using placebos or case cross-over studies. There are some studies with strong evidence of *Kampo* medicines' utility in other clinical faculties. Yokukansan is effective for the behavioral and psychological symptoms of dementia [34]. Daikenchuto prevents postoperative ileus after abdominal surgery [35]. Goreisan prevents postoperative recurrence of some types of chronic subdural hematomas [36]. Like these clinical trials, a large prospective study for headache treatment using *Kampo* medicine is needed.

*Kampo* medicine has side effects. The major side effects are pseudoaldosteronism, interstitial pneumonia, liver dysfunction, and allergy. Of the three types of *Kampo* medicines we used, kakkonto contains kanzo, which sometimes causes pseudoaldosteronism. Since long-term use of kakkonto may result in pseudoaldosteronism, other appropriate treatment modalities, such as physical therapy for TTH, are essential. Also, laboratory tests, X-rays, and physical examinations should be considered appropriately.

Limitation of this study

First, the sample size was small, and this study was performed in a single hospital with a single arm. Second, we did not compare to the control arm, so the true therapeutic effects of *Kampo* medicine were unknown. Third, the follow-up period differed for each patient, and side effects can occur in the long term. Therefore, we should follow up with the patients carefully. Fourth, we could not check the medication compliance rate of *Kampo* medicine other than the first month because some patients' headache diaries became less accurate as their symptoms improved. Finally, it was difficult to assess whether the improvement was due to the Western medication, a placebo effect, or spontaneous remission. Further studies using a control arm and placebo are considered in future research.

## Conclusions

The hybrid medication strategy of *Kampo* and Western medicines for CDH is safe and effective in terms of both acute and prophylactic medications with rapid efficacy. The decrease of HIT-6, MHD, MMD, and AMD, especially in EM+TTH and MOH patients, was observed. Goreisan, goshuyuto, and kakkonto can be used as alternative medicines for CDH treatment combined with Western medicine. Further studies are needed to establish the efficacy of *Kampo* medicine for headaches.

## References

[1] Couch JR (2011). Update on chronic daily headache. Curr Treat Options Neurol.

[2] Thorlund K, Toor K, Wu P (2017). Comparative tolerability of treatments for acute migraine: a network meta-analysis. Cephalalgia.

[3] Buse DC, Greisman JD, Baigi K, Lipton RB (2019). Migraine progression: a systematic review. Headache.

[4] Katsuki M, Yamagishi C, Matsumori Y (2022). Questionnaire-based survey on the prevalence of medication-overuse headache in Japanese one city-Itoigawa study. Neurol Sci.

[5] Biyajima M, Satoh S, Morikawa T (2022). Bromisoval-induced bromism with status epilepticus mimicking Wernicke's encephalopathy: report of two cases. BMC Neurol.

[6] Silberstein SD (2015). Preventive migraine treatment. Continuum (Minneap Minn).

[7] Headache Clinical Practice Guideline Development Committee (2021). Clinical Practice Guideline for Headache Disorders 2021. Clinical Practice Guideline for Headache Disorders.

[8] Katsuki M, Narita N, Matsumori Y, Ishida N, Watanabe O, Cai S, Tominaga T Kampo (Japanese herbal) medicine for primary headache as an acute treatment -a retrospective investigation in Kesennuma City Hospital during five years [PREPRINT]. J Neurosurg Kampo Med.

[9] Odaguchi H, Wakasugi A, Ito H, Shoda H, Gono Y, Sakai F, Hanawa T (2006). The efficacy of goshuyuto, a typical Kampo (Japanese herbal medicine) formula, in preventing episodes of headache. Curr Med Res Opin.

[10] Ishida K (2013). [Kampo medicines as useful therapeutic agents in clinical practice of neurology: case reports &amp; representative medicines]. Rinsho Shinkeigaku.

[11] Sanno N, Kawashima A, Ishii Y, Matsuno A, Morita A (2016). [Efficacy of the Kampo medicine in prevention of classical migraine] (Japanese). J Neurosurg Kampo Med.

[12] Katsuki M, Kawamura S, Kashiwagi K, Koh A (2021). Medication overuse headache successfully treated by Japanese herbal Kampo medicine, yokukansan. Cureus.

[13] Matsuda T (2019). [Hybrid treatment for migraine headaches using a combination of herbal and Western medicines] (Japanese). Japanese J Headache.

[14] (2018). Headache Classification Committee of the International Headache Society (IHS) the International Classification of Headache Disorders, 3rd edition. Cephalalgia.

[15] Kawamura T (2020). [Kampo medicine in neurosurgery] (Japanese). No Shinkei Geka.

[16] Shibata Y, Ishiyama S (2020). [An analysis of the meteorogical factors influencing climate-related headache and the clinical effects of goreisan] (Japanese). J Neurosurg Kampo Med.

[17] Shibata Y (2018). [Clinical analysis of the patients with primary headache in whom goreisan is effective] (Japanese). J Neurosurg Kampo Med.

[18] Katsuki M, Kawamura S, Kashiwagi K, Koh A (2021). Medication overuse headache successfully treated by three types of Japanese herbal Kampo medicine. Cureus.

[19] Shibata Y (2019). [Clinical analysis of the patients with tension-type headache in whom kakkontou is effective] (Japanese). J Neurosurg Kampo Med.

[20] Yarnell E (2017). Herbal medicine and migraine. Altern Complement Ther.

[21] Nakayama T, Tawara F, Miyano N (2016). [Aiming of higher-rank of treatment with Kampo medicine, toward the female medicine (Sterility, hyperemesis graviderm and climacteric symptom] (Japanese). J Japan Soc Menopause Women’s Heal.

[22] Noguchi T (2010). [Therapeutic effect of Goreisan for headache accompanying hemodialysis] (Japanese). Sci Kampo Med.

[23] Mukamal KJ, Wellenius GA, Suh HH, Mittleman MA (2009). Weather and air pollution as triggers of severe headaches. Neurology.

[24] Yasui H (2006). [Efficacy of ‘Poria Powder with Five Herbs’ for Headache: An epidemiological research study on the relationship between chronic headache and atmospheric depression] (Japanese). J Kampo, Acupunct Integr Med.

[25] Scheffler A, Schenk H, Wurthmann S, Nsaka M, Kleinschnitz C, Glas M, Holle D (2021). CGRP antibody therapy in patients with drug resistant migraine and chronic daily headache: a real-world experience. J Headache Pain.

[26] Kosinski M, Bayliss MS, Bjorner JB (2003). A six-item short-form survey for measuring headache impact: the HIT-6. Qual Life Res.

[27] Rothman KJ (1990). No adjustments are needed for multiple comparisons. Epidemiology.

[28] Voigt AW, Gould HJ 3rd (2016). Chronic daily headache: mechanisms and principles of management. Curr Pain Headache Rep.

[29] Silberstein S, Lipton R (2001). Chronic daily headache, including transformed migraine, chronic tension-type headache and medication overuse. Wolff’s Headache and Other Head Pain.

[30] Trucco M, Meineri P, Ruiz L, Gionco M (2010). Medication overuse headache: withdrawal and prophylactic therapeutic regimen. Headache.

[31] (1993). Guidelines and recommendations for the treatment of migraine. Italian Society for the Study of Headache (SISC). Funct Neurol.

[32] Couch JR (2011). Amitriptyline in the prophylactic treatment of migraine and chronic daily headache. Headache.

[33] Barbanti P, Egeo G, Aurilia C (2022). Fremanezumab in the prevention of high-frequency episodic and chronic migraine: a 12-week, multicenter, real-life, cohort study (the FRIEND study). J Headache Pain.

[34] Matsunaga S, Kishi T, Iwata N (2016). Yokukansan in the treatment of behavioral and psychological symptoms of dementia: an updated meta-analysis of randomized controlled trials. J Alzheimers Dis.

[35] Itoh T, Yamakawa J, Mai M, Yamaguchi N, Kanda T (2002). The effect of the herbal medicine dai-kenchu-to on post-operative ileus. J Int Med Res.

[36] Fujisawa N, Oya S, Yoshida S, Tsuchiya T, Nakamura T, Indo M, Matsui T (2021). A prospective randomized study on the preventive effect of Japanese herbal kampo medicine goreisan for recurrence of chronic subdural hematoma. Neurol Med Chir (Tokyo).

